# Management of Inflammatory Internal Root Resorption with Biodentine and Thermoplasticised Gutta-Percha

**DOI:** 10.1155/2015/452609

**Published:** 2015-10-22

**Authors:** Girish Umashetty, Upendra Hoshing, Suvarna Patil, Nishant Ajgaonkar

**Affiliations:** Department of Conservative Dentistry & Endodontics, Vasantdada Patil Dental College Kavalapur, Sangli, Maharashtra 416306, India

## Abstract

Internal root resorption is a chronic inflammatory process initiated within the pulp space with the loss of dentin. This condition demands a comprehensive understanding of the pathologic process, so as to identify the cause and arrest the resorptive phenomena. It is a rare occurrence, asymptomatic, with slow progression, detected through routine radiographic examination, where it appears as a radiolucent lesion. This paper reports a clinical case of inflammatory internal root resorption in the premolar tooth. Because it is asymptomatic, internal root resorption needs an early diagnosis in order to institute the endodontic treatment before the process compromises the remaining mineralized structures of the tooth. Biodentine was used to reinforce the weaker structures in the root. Thermoplasticised gutta-percha was used to completely obturate the defect. Ten-month follow-up showed arrest of internal root resorption.

## 1. Introduction

Resorption is a condition associated with either a physiologic or a pathologic process resulting in a loss of dentin, cementum, and/or bone [1]. Root resorption may occur after various injuries, including mechanical, chemical, or thermal injury. Internal resorption is established after necrosis of odontoblasts and it is associated with chronic partial pulp inflammation and partial pulp necrosis [2]. It can be associated with many factors such as partial removal of the pulp, caries, trauma, pulp capping with calcium hydroxide or pulpotomy, extreme heat, and a cracked tooth. These factors stimulate the pulp tissue, thus initiating inflammatory processes and then some undifferentiated cells of the pulp convert themselves to osteoclasts or macrophages, which results in dentinal resorption [3]. In order to control the internal root resorption, it is necessary to treat the root canal aiming to remove all the pulp tissue and achieve a better sealing. Combined treatment plan using bioactive materials like Biodentine along with thermoplasticised gutta-percha obturation will not only give a three-dimensional obturation but also induce remineralization and healing [4].

## 2. Case Report

**Figure 1:**
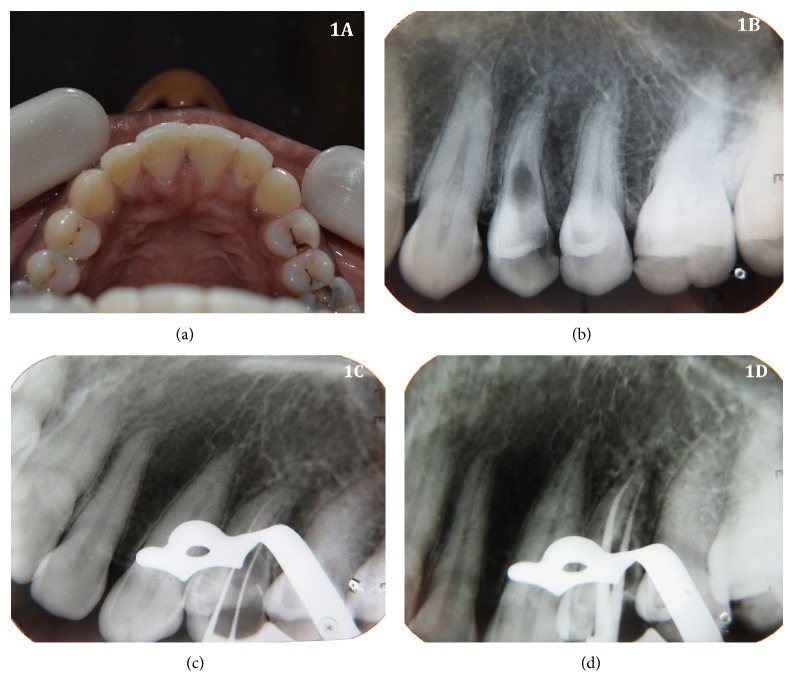
(a) Clinical photograph of maxillary arch occlusal view. (b) Preoperative radiograph. (c) Working length determination radiograph. (d) Master cone selection radiograph.

A 30-year-old female patient reported to the Department of Conservative Dentistry & Endodontics, Vasantdada Patil Dental College, with a chief complaint of food lodgement in maxillary left first premolar tooth (number 24). Patient gave history that the tooth was decayed for the past 5-6 years and denied any past or recent episode of trauma. Clinical examination revealed presence of distoocclusal caries in tooth number 24 (Figure 1(a)) and absence of draining sinus, swelling, and tenderness to percussion. Medical history was noncontributory. Radiographic examination by an intraoral periapical radiograph revealed distoocclusal caries in  tooth number 24 approaching pulp chamber (Figure 1(b)). An oval shaped radiolucency within the root periphery of tooth number 24 at the junction of coronal and middle one-third of the root was observed, suggestive of internal root resorption, which was confirmed by taking multiple angulated radiographs. It is worth highlighting that the patient had no pain, normal space on the periodontal ligament, and no mobility of the tooth. Vitality testing of the involved tooth with dry ice (R C Ice; Prime Dental Products India) and electronic pulp stimulation (Parkel Electronics Division, Farmingdale, NY) gave a positive response. From the clinical and radiographic findings, a diagnosis of inflammatory internal root resorption in  tooth number 24 was made and endodontic treatment of tooth number 24 was suggested to the patient.

**Figure 2:**
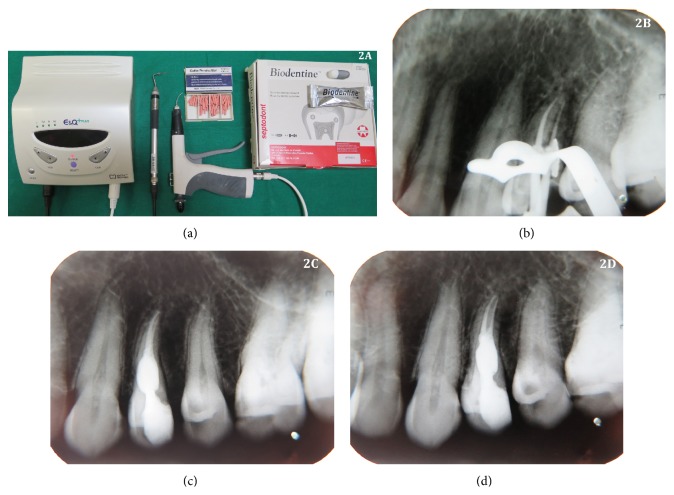
(a) E & Q (META BIOMED) system apparatus (pen, gun, and gutta-percha bar) and Biodentine capsule (Septodont, Saint-Maur-des-Fossés, France). (b) Confirmatory radiograph after placement of Biodentine. (c) Immediate postoperative radiograph. (d) Ten-month follow-up radiograph.

Local anesthesia (2% lignocaine with 1 : 80000 adrenaline) was administered and tooth number 24 was isolated under rubber dam. Removal of caries was done followed by access cavity preparation with high speed air turbine with careful attention to the direction of the diamond point to prevent accidental perforation. Canals were thoroughly irrigated with normal saline followed by 3% sodium hypochlorite. The working length of the tooth was measured first manually and then by Root ZX apex locator (J. Morita Mfg. corp., Kyoto, Japan) which was confirmed radiographically (Figure 1(c)). Biomechanical preparation was done of 24 using ProTaper nickel-titanium rotary instruments (Dentsply Maillefer) in a crown-down technique till F3 file. Root canals were copiously irrigated with 3% sodium hypochlorite solution using nontraumatic plastic tips of EndoActivator (Dentsply) for achieving a complete chemomechanical debridement. A calcium hydroxide dressing (RC CAL, Prime Dental Products, India) was placed for a period of 30 days, with change of the medication after 15 days. A small cotton pallet was placed and the access cavity was temporarily sealed with Cavit (3M ESPE, Saint Paul, MN). After 30-day interval, the root canal was reentered and irrigated with normal saline and hand filing with number 15 K file alternately to remove the calcium hydroxide medicament, followed by 3% NaOCl, 17% EDTA, and normal saline used as the final irrigant. As the internal resorption defect periphery was very thin radiographically, absence of any perforation during root canal instrumentation was confirmed with dried sterile absorbent paper points. Master cone selection was done and confirmed radiographically (Figure 1(d)). Biodentine capsule (Septodont, Saint-Maur-des-Fossés, France) (Figure 2(a)) was mixed according to manufacturer's instructions and capsule material which had good creamy consistency was inserted into the resorption cavity using a nonsurgical MTA carrier (Micro Apical Placement System, Produits Dentaires, Vevey, Switzerland) and was condensed laterally against the walls of resorption cavity with root canal spreaders (Dentsply Maillefer) to reinforce the weakened tooth structure of the resorptive defect (Figure 2(b)). After ensuring complete set of the material (setting time 12 mins), the remaining resorptive defect was obturated using thermoplasticised gutta-percha technique. Obturation was done with a down pack of gutta-percha using E & Q pen (META BIOMED) (Figure 2(a)) with AH plus sealer (Dentsply). The remainder of canal was backfilled with an E & Q gun using thermoplastic gutta-percha. Warm gutta-percha at the orifice was vertically compacted by using appropriate sized pluggers. After completion of root canal treatment, the tooth was restored using posterior resin composite (P60; 3M Dental Products, Saint Paul, MN). Immediate postoperative radiograph showed dense obturation in the resorptive defect (Figure 2(c)). After ten-month follow-up, the patient was asymptomatic demonstrating a functional tooth number 24. Radiographic examination revealed stoppage of resorptive process with intact dense obturation (Figure 2(d)). The patient was advised with full-coverage porcelain crown.

## 3. Discussion

Internal root canal inflammatory resorption involves a progressive loss of intraradicular dentin without adjunctive deposition of hard tissues adjacent to the resorptive sites. It is frequently associated with chronic pulpal inflammation, and bacteria might be identified from the granulation tissues when the lesion is progressive to the extent that it is identifiable with routine radiographs [5]. As it is asymptomatic, it is detected coincidentally through routine radiographs. Internal resorption only occurs when the predentin adjacent to the site of chronic inflammation is lost as a result of trauma [6] or other unknown etiologic factors. In a study including 27 patients, trauma is the most common etiological factor (43%), followed by carious lesions (25%) [7]. The condition might go unnoticed until the lesion has advanced significantly, resulting in a perforation [8] or symptoms of acute or chronic apical periodontitis after the entire pulp has undergone necrosis and the pulp space has become infected. Hence, root canal treatment must be initiated as soon as possible once an inflammatory resorptive lesion is detected to prevent further hard tissue loss and eventual root perforation [9].

In the present case, considering the patient's age, periodontal status, resorption location, absence of perforations, and resistance of remaining root hard tissue, a nonsurgical endodontic treatment along with reinforcement by bioactive material like Biodentine remains the treatment of choice. The shape of the resorption defect usually makes it inaccessible to direct mechanical instrumentation [10]. So the use of ultrasonic devices like EndoActivator activates and facilitates the penetration of the irrigation solution of hypochlorite to all the areas of the root canal system [9], thus achieving complete chemomechanical debridement. The use of calcium hydroxide as an interappointment dressing helps to control the bleeding, maximizes the effect of disinfection procedures, maintains alkalinity, and necrotizes residual pulp tissue [11]. Remnants of calcium hydroxide may affect bonding of Biodentine to dentin; hence, its complete removal is necessary [12]. Considering the thin and weakened tooth structure in the resorptive defect, a bioactive material (ProRoot MTA, Biodentine) was needed to reinforce the tooth and thereby enhance the prognosis of the tooth. Biodentine was used because it has some features which are superior to MTA; for example, its consistency is better suited to the clinical use than MTA's and Biodentine does not require two-step obturation as in the case of MTA because of its faster setting time of about 12 minutes [12]. Biodentine powder is mainly composed of tricalcium silicate, calcium carbonate, and zirconium oxide as the radiopacifier, whilst Biodentine liquid contains calcium chloride as the setting accelerator and water reducing agent. It has the capacity to develop watertight interfaces both with dental structures and with adhesive systems [12]. Biodentine shows deposition of apatitic structures which might increase the marginal sealing of the material [12]. About the root canal filling, the obturating material needs to be flowable to seal the resorptive defect. Thermoplastic gutta-percha technique seems to give the best results when the canal walls are respected [13, 14]. The success or failure of therapy should be followed up clinically and by radiographic control.

## 4. Conclusion

The early diagnosis and therapy are very important in order to stop the resorption process. It is imperative to initiate endodontic treatment as soon as possible to arrest the progression of the resorptive process and to prevent further weakening of tooth structure. Success in management of a case of internal resorption depends on early detection, appropriate treatment planning, removal of inflammatory pulp tissue, reinforcement of weaker tooth structure, and a three-dimensional obturation.
